# Occupational Exposure to *Staphylococcus aureus* and *Enterococcus* spp. among Spray Irrigation Workers Using Reclaimed Water

**DOI:** 10.3390/ijerph110404340

**Published:** 2014-04-17

**Authors:** Rachel E. Rosenberg Goldstein, Shirley A. Micallef, Shawn G. Gibbs, Xin He, Ashish George, Amir Sapkota, Sam W. Joseph, Amy R. Sapkota

**Affiliations:** 1 Maryland Institute for Applied Environmental Health, School of Public Health, University of Maryland, College Park, MD 20742, USA; E-Mails: rerosenb@umd.edu (R.E.R.G); smicall@umd.edu (S.A.M); ashishbg88@gmail.com (A.G.); amirsap@umd.edu (A.S.); swj@umd.edu (S.W.J.); 2 Department of Plant Science and Landscape Architecture, University of Maryland, College Park, MD 20742, USA; 3 Center for Food Safety and Security Systems, University of Maryland, College Park, MD 20742, USA; 4 Department of Environmental, Agricultural and Occupational Health, College of Public Health, University of Nebraska Medical Center, Omaha, NE 68198, USA; E-Mail: sgibbs@unmc.edu; 5 Department of Epidemiology and Biostatistics, School of Public Health, University of Maryland, College Park, MD 20742, USA; E-Mail: xinhe@umd.edu; 6 Department of Cell Biology and Molecular Genetics, University of Maryland, College Park, MD 20742, USA

**Keywords:** antibiotic-resistance, *Staphylococcus aureus*, enterococci, spray irrigation, occupational exposure

## Abstract

As reclaimed water use expands, it is important to evaluate potential occupational health risks from exposure to this alternative water source. We compared odds of colonization with methicillin-resistant *Staphylococcus aureus* (MRSA), methicillin-susceptible *S. aureus* (MSSA), vancomycin-resistant enterococci (VRE), and vancomycin-susceptible enterococci (VSE) between spray irrigation workers using reclaimed water and office worker controls. Nasal and dermal swabs from 19 spray irrigation workers and 24 office worker controls were collected and analyzed for MRSA, MSSA, VRE, and VSE. Isolates were confirmed using standard biochemical tests and polymerase chain reaction assays. Antimicrobial susceptibility testing was performed by Sensititre^®^ microbroth dilution. Data were analyzed by two-sample proportion, chi-square, Fisher’s exact tests, and logistic regression. No MRSA or VRE were detected in any samples. MSSA was detected in 26% and 29% of spray irrigators and controls, respectively. VSE was detected in 11% and 0% of spray irrigation workers and controls, respectively. The adjusted odds of MSSA, multidrug-resistant MSSA, and either MSSA or VSE colonization were greater among spray irrigation workers, however results were not statistically significant. Future studies with larger sample sizes are needed to further evaluate this relationship.

## 1. Introduction

Between 5%–6% of municipal wastewater effluent, approximately 2.22 billion gallons per day, is reclaimed and reused in the United States [1]. Landscape irrigation is one of the most common uses of reclaimed water in the USA, making up 18% of all water reused across the country [2]. Although irrigation with reclaimed water is increasing, limited data exist on the pathogens that may be present in reclaimed water, as well as the occupational health risks from exposure to this water source [3,4,5,6]. Some studies have identified several bacterial and parasitic species in reclaimed water [3,4,7]. Methicillin-resistant *Staphylococcus aureus* (MRSA) and vancomycin-resistant enterococci (VRE) have both been identified in wastewater throughout the treatment process, including final effluent in the U.S. and Europe [7,8,9,10,11,12]. Several studies have suggested that wastewater, and the reuse of wastewater, could be a source of exposure to these antibiotic-resistant bacteria, as well as other human pathogens, in the community [7,9]. As rates of antibiotic-resistant bacterial infections in hospitals and the community continue to rise [13], including infections with MRSA and VRE, it is important to evaluate whether reclaimed water could serve as a potential source of exposure to these microorganisms. 

*Staphylococcus aureus* is a bacterial pathogen that colonizes multiple body sites, most commonly the nostrils, and causes a number of infections, including skin and soft tissue infections, pneumonia, and septicemia [14]. MRSA and methicillin-susceptible *S. aureus* (MSSA) have been detected in air samples from a number of environments, including wastewater treatment plants [15]*.* Since these bacteria can be aerosolized from water and are capable of colonizing skin and soft tissues, exposure through inhalation is of concern, particularly among workers at both wastewater treatment plants and spray irrigation sites who use or come in contact with the reclaimed water.

Enterococci are opportunistic pathogens that can cause urinary tract infections, bacteremia, and endocarditis [16]. Between 2006 and 2007, 13% of hospital infections were caused by enterococci, and approximately 30% of these infections were VRE [17]. VRE and vancomycin-susceptible enterococci (VSE) have been detected in wastewater in both raw influent and treated effluent, as well as wastewater bioaerosols [8,10,11,15,18,19,20]. Although the exact sources of community-acquired VRE and VSE infections remain unclear, animals, food, and wastewater have been suggested as important environmental reservoirs [21,22].

If *S. aureus* or enterococci survive wastewater treatment and distribution to reuse sites, spray irrigation workers using reclaimed water could be exposed to these organisms through dermal contact or inhalation [23]. To our knowledge, no previous studies have evaluated the risk for occupational exposure to antibiotic-resistant bacteria from reclaimed water [2,5]. In the present study, we compared MRSA, MSSA, VRE, and VSE colonization among spray irrigation workers using reclaimed water and office worker controls in the Mid-Atlantic region of the United States.

## 2. Experimental Section

### 2.1. Study Site

The reclaimed water spray irrigation site included in this study is located in the Mid-Atlantic region of the USA. The site was chosen based on the willingness of the site operator to participate in the study. This site receives treated wastewater from a tertiary wastewater treatment plant (Mid-Atlantic WWTP1) [7,12] that serves an urban area, processing 681,390 m^3^/day of wastewater, with a peak capacity of 1.51 million m^3^/day. The incoming wastewater (influent) at Mid-Atlantic WWTP1 includes domestic and hospital wastewater, and the following treatment steps are employed at the plant: screens, primary clarifier, activated sludge reactors, secondary clarifier, sand filters, chlorination, dechlorination and discharge. At the time of the study, the chlorination dose was 2–3 mg/L, followed by dechlorination with sodium bisulfite such that the chlorine residual in the effluent was <0.1 mg/L. 

The effluent (discharge) from Mid-Atlantic WWTP1 is sent to our spray irrigation study site through an enclosed pipe. Once the treated wastewater reaches the spray irrigation site, it passes through an aluminum screen and is then treated with 254 nanometer wavelength ultraviolet (UV) radiation bulbs that produce a minimum of 30,000 microwatt seconds per square centimeter. After UV treatment, the water is pumped into an open-air storage pond at a rate of 230,000 gallons per day with a peak capacity of 4 million gallons. Based on turf irrigation needs, water from the storage pond is pumped to spray heads throughout the site. Spray irrigation workers also carry backpack spray systems to irrigate additional areas. The spray irrigation site employs eight full-time employees and approximately 22 seasonal employees each year.

### 2.2. Subject Selection

This study was approved by the University of Maryland College Park, Institutional Review Board, IRB Protocol 09-0211. A total of 43 subjects were enrolled in the study; 19 spray irrigation workers from the study site who were occupationally exposed to reclaimed water, and 24 office worker controls from an academic work setting who were not exposed to reclaimed water or healthcare settings on the job. Study subjects were selected through a convenience sample based on employment status. Office worker controls were matched by age (±2 years), sex, and race to the spray irrigation workers and recruited into the study in person and over email. Individuals were excluded from participation if they reported a nosebleed three days prior to sample collection to avoid dislodging blood clots.

### 2.3. Survey

Participants were asked to complete a short survey containing questions related to sociodemographics, as well as questions related to risk factors associated with MRSA colonization and previous MRSA diagnosis. The survey also asked participants about previous work in healthcare facilities and household members who work in healthcare facilities, because *S. aureus* nasal colonization rates among healthcare workers is greater than the 20%–30% colonization rate found in the general population [24,25]. The survey was filled out by participants on site at each sampling event.

### 2.4. Sample Collection

A total of 94 nasal (48 from spray irrigation workers and 46 from office worker controls) and 94 dermal swab samples (48 from spray irrigation workers and 46 from office worker controls) were collected between August 2009 and February 2011 when the irrigation spray heads were in use. Participants were sampled at multiple time points when possible. On average, participants were each sampled 2.19 times. Nasal swabs were collected using Liquid Stuart Medium Transport swabs (Copan, Brescia, Italy). The swab was inserted approximately 1.25 cm into the participant’s right nostril and gently rotated five times on the inside wall of the nostril [23,26]. Dermal swabs were also collected using the same type of swabs. An approximately five-by-five cm area of the participant’s right forearm was swabbed by rolling the swab back and forth 15 times [27]. All samples were transported to the laboratory at 4 °C and processed within 24 h.

### 2.5. Isolation

All media was obtained from Becton, Dickinson and Company (Franklin Lakes, NJ, USA). Nasal and dermal swabs were streaked onto Baird Parker agar for isolation of total *S. aureus* and Enterococcosel agar for isolation of total *Enterococcus* spp. Baird Parker agar plates were incubated at 37 °C for 24 h, while Enterococcosel agar plates were incubated at 41 °C for 24 h. Resulting black colonies with halos on Baird Parker, and colonies with a black precipitate on Enterococcosel agar were considered presumptive *S. aureus* and enterococci, respectively. These colonies were purified on Brain Heart Infusion agar and archived in Brucella broth with 15% glycerol at −80 °C. *S. aureus* ATCC 43300 and *Enterococcus* *faecalis* ATCC 29212 were used as positive controls and phosphate buffered saline was used as a negative control throughout the isolation process.

### 2.6. Confirmation

*S. aureus* were identified using the Gram stain, the coagulase test (Becton, Dickinson and Company), and the catalase test. For confirmation of *S. aureus* and MRSA differentiation, the *S. aureus*-specific *nuc* gene, the MRSA-specific *mecA* gene, and a 16S rDNA internal control were PCR amplified in a multiplex reaction as described previously [7,28]. DNA extraction was performed using the MoBio UltraClean® Microbial DNA Isolation Kit (Mo Bio Laboratories, Carlsbad, CA, USA) per the manufacturer’s recommendations. Briefly, PCR amplification consisted of an initial denaturing step of 95 °C for 3 min, followed by 34 cycles of denaturing at 94 °C for 30 s, annealing at 55 °C for 30 s, and extension at 72 °C for 30 s, with a final extension at 72 °C for 5 min. *S. aureus* ATCC 43300 was used as a positive control for PCR amplification of *nuc* and *mecA* genes.

Enterococci were presumptively identified using the Gram stain, the catalase test, and by detection of pyrrolidonyl peptidase (pyr) activity (Remel, Lenexa, KS, USA). Confirmation was accomplished using a modified multiplex PCR assay previously described by Micallef *et al.* [29]. Genomic DNA from enterococci was extracted by heat lysis as described previously [29]. Briefly, the PCR reaction targeted the *ddl* genes of *Enterococcus faecalis* and *E. faecium*, the vanC1 and vanC2/3 genes of *E. gallinarum* and *E. casseliflavus*, and a 16S rDNA internal control [29]. PCR amplification consisted of an initial denaturing step of 95 °C for 3 min, followed by 35 cycles of denaturing at 94 °C for 30 s, annealing at 54 °C for 30 s, and extension at 72 °C for 30 s, with a final extension at 72 °C for 5 min. Positive controls used for PCR amplification were *E. faecalis* ATCC 51299, *E. faecium* ATCC 51559, *E. casseliflavus* ATCC 25788, and *E. gallinarum* ATCC 49573. Colonization among study participants was defined as MRSA, MSSA, VRE, or VSE isolated and confirmed from any swab sample (dermal or nasal) during the study period.

### 2.7. Antimicrobial Susceptibility Testing

Antimicrobial susceptibility testing was performed using the Sensititre® microbroth dilution system with GPN3F minimal inhibitory concentration (MIC) plates (Trek Diagnostic Systems Inc., Cleveland, OH, USA) in accordance with the manufacturer’s instructions on all PCR-confirmed *S. aureus* (n = 97) and *Enterococcus* spp. (n = 20) isolates. Briefly, overnight cultures were transferred to sterile demineralized water (Trek Diagnostic Systems) to achieve a 0.5 McFarland standard. Then, 30 μL of the suspension for *S. aureus* and 50 μL of the suspension for enterococci was transferred to sterile cation-adjusted Mueller Hinton broth (Trek Diagnostic Systems), and 50 μL of the broth solution was then dispensed into the GPN3F MIC plates (Trek Diagnostic Systems Inc.) with the following antibiotics: erythromycin (ERY; 0.25–4 μg/mL), clindamycin (CLI; 0.12–2 μg/mL), quinupristin/ dalfopristin (SYN; 0.12–4 μg/mL), daptomycin (DAP; 0.25–8 μg/mL), vancomycin (VAN; 1–128 μg/mL), tetracycline (TET; 2–16 μg/mL), ampicillin (AMP; 0.12–16 μg/mL), gentamicin (GEN; 2–16, 500 μg/mL), levofloxacin (LEVO; 0.25–8 μg/mL), linezolid (LZD; 0.5–8 μg/mL), ceftriaxone (AXO; 8–64 μg/mL), streptomycin (STR; 1,000 μg/mL), penicillin (PEN; 0.06–8 μg/mL), rifampin (RIF; 0.5–4 μg/mL), gatifloxacin (GAT;1–8 μg/mL), ciprofloxacin (CIP; 0.5–2 μg/mL), trimethoprim/sulfamethoxazole (SXT; 1/19–4/76 μg/mL), and oxacillin + 2%NaCl (OXA+; 0.25–8 μg/mL). Plates were incubated at 37 °C and read after 18–24 h. *Enterococcus faecalis* ATCC 29212 and *S. aureus* ATCC 29213 were used as quality control strains. MICs were recorded as the lowest concentration of an antimicrobial that completely inhibited bacterial growth [30]. Resistance breakpoints published by the Clinical and Laboratory Standards Institute were used [30]. Multidrug resistance (MDR) was defined as resistance to two or more classes of antibiotics.

### 2.8. Statistical Analyses

Descriptive statistics were reported including the percentages of study participants that were positive for MSSA and VSE by worker classification. Differences in sociodemographic variables between spray irrigation workers and controls were compared using the chi-square or Fisher’s exact test. Statistical analyses of antibiotic resistance data were limited to MSSA (n = 32) and VSE (n = 3) isolates expressing unique antimicrobial resistance profiles; this allowed us to reduce bias that could be introduced by including possible clones. A two-sample *t*-test was used to compare the number of antibiotics that isolates expressed resistance against. Logistic regression models were used (1) to determine if the odds of ever being colonized with the bacteria of interest were different for spray irrigation workers compared to controls, while controlling for education, duration of job, yearly income, and current smoking status; and (2) to conduct analyses of spray irrigation worker odds of colonization. A generalized linear mixed effects model (GLMM) was used to evaluate the odds of being colonized with *S. aureus* over time by occupational status. In all cases, *p*-values of ≤0.05 were defined as statistically significant. All statistical analyses were performed using Stata/IC 10 (StatCorp LP, College Station, TX, USA) or SAS 9.2 (SAS Inc., Cary, NC, USA).

## 3. Results and Discussion

### 3.1. Results

The participation rate for the study was 88% (43/49). Participants ranged in age from 17 to 66 years and both spray irrigation workers and office worker controls were composed largely of Caucasian males (Table 1). The mean age of the spray irrigation workers and controls was 34 and 33 years, respectively. None of the participants in either group reported previous diagnoses of MRSA. Of the sociodemographic variables collected from participants, there were a few significant differences between spray irrigation workers and controls. Education levels and yearly income differed by exposure group (*p*<0.001; *p* = 0.01). Spray irrigation workers reported “currently smoking” (*p* = 0.002) and “smoking more than 100 cigarettes in the past six months” (*p* < 0.001) more than the controls (Table 1). Slightly more controls reported either personally having worked in a healthcare setting or having a household member who had worked in a healthcare setting, but these differences were not statistically significant. 

**Table 1:** Comparison of participant characteristics between spray irrigation workers and office worker controls **^a^**.

Variable	n (%)		*p*-value	
	Spray Irrigation Workers	Office Worker Controls		
Total	19	24		
*Age (years)*			0.41	
≤17	3 (16)	0 (0)		
18–19	2 (11)	2 (8)		
20–30	4 (21)	10 (42)		
31–41	5 (26)	6 (25)		
42–56	4 (21)	5 (21)		
>56	1 (5)	1 (4)		
*Gender*			1.00	
Male	18 (95)	23 (96)		
Female	1 (5)	1 (4)		
*Race*			0.58	
Caucasian	17 (90)	23 (96)		
Other	2 (10)	1 (4)		
*Education*			<0.001	
Less than high school	1 (5)	0 (0)		
High school	12 (63)	0 (0)		
Associate	2 (11)	0 (0)		
College	4 (21)	24 (100)		
*Yearly income ($1,000s)*			0.01	
<15	10 (56)	5 (21)		
15–25	3 (17)	6 (25)		
25–35	2 (11)	1 (4)		
35–50	2 (11)	1 (4)		
>50	1 (6)	11 (46)		
*Duration in job*			0.22	
≤1 month	2 (10.5)	1 (4)		
>1 month–≤6 months	6 (31.5)	3 (12.5)		
>6 months–≤2 years	3 (16)	5 (21)		
>2–≤5 years	5 (26)	5 (21)		
>5–≤ 20 years	3 (16)	5 (21)		
≥20 years	0 (0)	5 (21)		
*Currently smoke*			0.002	
Yes	10 (53)	2 (8)		
No	9 (47)	22 (92)		
*Smoke more than 100 cigarettes in past 6 months*			<0.001	
Yes	9 (47)	0 (0)		
No	10 (53)	24 (100)		
*Personally worked in healthcare setting*			1.00	
Yes	3 (16)	4 (17)		
No	16 (84)	20 (83)		
*Household member worked in healthcare setting*			0.69	
Yes	6 (32)	9 (37.5)		
No	13 (68)	15 (62.5)		

Note: **^a^** Differences between spray irrigation workers and controls were analyzed using Fisher’s exact test for all analyses except “Household Member Worked in Healthcare Setting” which was analyzed using the chi-square test.

#### 3.1.1. Presence of MRSA and MSSA

**Figure 1:**
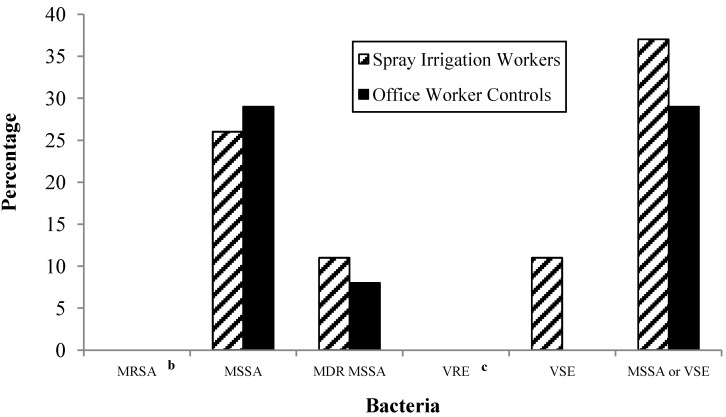
Prevalence of methicillin-resistant *Staphylococcus aureus* (MRSA), methicillin-susceptible *S. aureus* (MSSA), multidrug-resistant (MDR) MSSA, vancomycin-resistant enterococci (VRE), vancomycin-susceptible enterococci (VSE), and MSSA or VSE among spray irrigation workers (n = 19) and office worker controls (n = 24) **^a^**.

No MRSA was detected in any of the nasal or dermal swabs collected from the spray irrigation workers or controls. MSSA was recovered from 28% (12/43) of all study participants. Twenty-six percent (5/19) of spray irrigation workers had nasal swabs that were positive for MSSA during at least one sampling event (Figure 1). Among controls, 29% (7/24) were positive for MSSA in nasal swabs during at least one sampling event. We did not detect MSSA in any spray irrigation worker dermal swab samples, but 8% (2/24) of controls were MSSA-positive based on dermal swab samples alone. The probability of ever having been colonized with MSSA from either sample type was not significantly different between spray irrigation workers and controls (*p* = 0.84).

Notes:^** a**^ Prevalence is defined as a nasal or dermal swab from a participant ever being positive for the target organism. Participants were swabbed an average of 2.19 times. **^b^** No MRSA was detected in any samples. **^c^** No VRE was detected in any samples.

#### 3.1.2. Presence of VRE and VSE

VRE was not detected in any nasal or dermal swab samples from the spray irrigation workers or controls (Figure 1). A greater proportion of spray irrigation workers were colonized with VSE compared to controls (11% *vs.* 0%; *p* = 0.19).

#### 3.1.3. Presence of Either of the Target Bacteria in Swab Samples

Thirty-three percent (14/43) of all participants were colonized with either MSSA or VSE. A greater proportion of spray irrigation workers compared to controls were colonized with at least one of the bacteria of interest (*p* = 0.30) (Figure 1).

#### 3.1.4. Antibiotic Resistance Patterns

**Figure 2:**
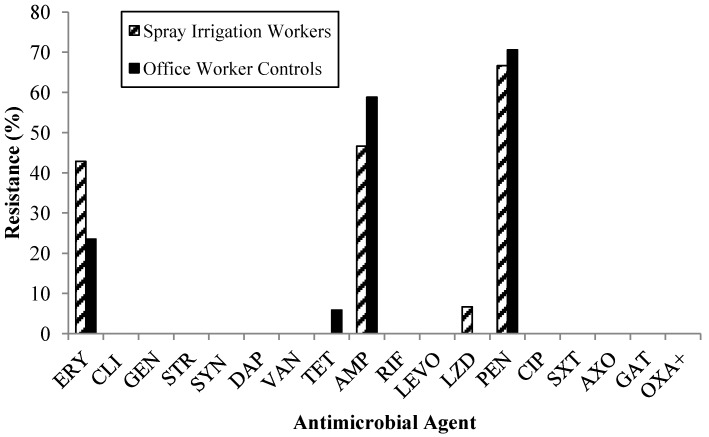
Percent resistance to antimicrobial agents observed among MSSA isolates recovered from spray irrigation worker and office worker control nasal and dermal swabs.

Notes: ERY = erythromycin; CLI = clindamycin; SYN = quinupristin/dalfopristin; DAP = daptomycin; VAN = vancomycin; TET = tetracycline; AMP = ampicillin; GEN = gentamicin; LEVO = evofloxacin; LZD = linezolid (LZD; 0.5–8 μg/mL), AXO = ceftriaxone; STR = streptomycin; PEN = penicillin; RIF = rifampin; GAT = gatifloxacin; CIP = ciprofloxacin; SXT = trimethoprim/sulfamethoxazole; OXA+ = oxacillin.

In total, 97 MSSA isolates were recovered from nasal and dermal swabs: 57 isolates from spray irrigation workers, and 40 from controls. However, statistical analyses concerning antibiotic resistance patterns among these isolates were limited to 32 isolates that could be confirmed as unique using phenotypic analyses (15 from spray irrigation workers; 17 from controls). Isolates were resistant to a variety of the 18 antibiotics tested. A greater percentage of spray irrigation workers compared to controls were colonized with MDR MSSA (11% *vs.* 8.3%) (*p* = 0.40) (Figure 1) and a greater percentage of MSSA isolates from spray irrigation worker swabs were resistant to erythromycin (*p* = 0.13) and linezolid (*p* = 0.13) (Figure 2). A greater percentage of MSSA isolates from office worker control swabs compared to spray irrigation worker swabs were resistant to tetracycline (*p* = 0.17) and ampicillin (*p* = 0.25) (Figure 2). MSSA isolates from spray irrigation workers’ nasal swabs were resistant to an average of 1.6 antibiotics compared to 1.47 antibiotics among control nasal swab MSSA isolates (*p* = 0.37). 

In total, 20 VSE isolates were isolated from two spray irrigation worker nasal swabs. Of the three isolates that were phenotypically unique, all three were resistant to rifampicin and all three were either resistant or intermediately resistant to quinupristin/dalfopristin.

#### 3.1.5. Impact of Occupational Exposure on Colonization

Our unadjusted and adjusted logistic regression models indicated that the odds of being colonized with MDR MSSA or either MSSA or VSE were greater among the spray irrigation workers compared to the controls; however, the differences were not statistically significant (Table 2). In the adjusted model, the odds of being colonized with MSSA were also greater among spray irrigation workers compared to controls but the difference was not statistically significant (Table 2). After adjusting for changes over time—in addition to education, duration of job, yearly income, and current smoking status—using the GLMM, there were no significant differences in the odds of colonization with any of the bacteria of interest (*p* = 0.75) (data not shown).

**Table 2:** Estimated odds ratios of ever being colonized with MSSA, MDR MSSA, or either MSSA or VSE, by occupational status.

	Unadjusted OR	95% CI	Adjusted OR *	95% CI
**MSSA**				
Spray irrigation worker	0.87	0.23, 3.34	1.40	0.09, 22.40
Office worker control				
**MDR MSSA**				
Spray irrigation worker	1.29	0.17, 10.15	7.01	0.13, 367.77
Office worker control	—			
**MSSA or VSE**				
Spray irrigation worker	1.42	0.39, 5.11	2.55	0.15, 44.15
Office worker control	—			

Note: * Confounders included in the adjusted model were: education, duration of job, yearly income, and current smoking status. Confounders were defined as a change in OR of ≥10%.

#### 3.1.6. Factors Impacting MSSA and VSE Colonization among Spray Irrigation Workers

The results of our logistic regression models focused only on spray irrigation workers showed that most of the variables used in our model did not have statistically significant effects on the odds of the spray irrigation workers being colonized with MSSA, MDR MSSA, VSE, or either MSSA or VSE (data not shown). However, spray irrigation workers, who reported either personally having worked in a healthcare setting or having a household member who had worked in a healthcare setting (n = 8) tended to be more likely to be colonized with either MSSA or VSE compared to those who did not report this type of exposure but the difference was not statistically significant (OR = 7.50; 95% CI 0.92–61.05).

### 3.2. Discussion

#### 3.2.1. MRSA and MSSA Prevalence

Based on the 2003–2004 National Health and Nutrition Examination Survey (NHANES), between 27.2% and 30% of the U.S. population are colonized with *S. aureus* [25]. It has also been estimated that 20%–30% of the general population is colonized in the nostrils with *S. aureus* [24,31]. Twenty-eight percent of all study participants in this study were nasally colonized with MSSA, which falls within the expected range of MSSA prevalence. Previous studies have identified low carriage rates of MRSA in the community, including a study by Gorwitz *et al.* that found that the prevalence of MRSA colonization in the U.S. is approximately 1.5% based on the 2003–2004 NHANES data [25]. Therefore, the finding that MRSA was not detected in any samples in the current study could be a factor of our small sample size. Also, the lack of any statistically significant differences in the odds of MSSA, MDR MSSA, and MSSA or VSE colonization between spray irrigation workers and office worker controls could also be due to our small sample size. To find a significant difference between these two groups we would have needed to enroll at least 3,453 spray irrigation workers and 3,453 controls. Our experience, and the anecdotal experience of others, however, has shown that gaining access to wastewater reclamation sites for research purposes and finding spray irrigation workers willing to participate is quite difficult, severely limiting the number of possible study participants in a study of this sort [32].

#### 3.2.2. VRE and VSE Prevalence

Community-associated VRE (CA-VRE) (defined as no previous hospital stay reported) is rarely reported in the USA. [22,33], however, the introduction of VRE into hospital settings from outside environments has been documented in both the U.S. and in other countries [33,34]. In a study by Stevenson *et al.* of rural U.S. hospitals, 22% of patients with positive VRE cultures had been in the hospital for under 48 h or were outpatients, prompting classification as a CA-VRE infection [33]. A 1999 study in Germany found a 0.9% prevalence of VRE among a “healthy” student population, not admitted to a hospital for infection with VRE [35]. Therefore, the fact that VRE was not detected in any of the current study’s participants could also be a factor of our limited sample size. 

*E. faecalis* and *E. faecium* have previously been isolated in small numbers from the upper respiratory tract; however, in a large-scale hospital study of VRE and MRSA colonization by Warren *et al.*, the majority of VRE-positive specimens were recovered from stool or rectal samples (87%) compared to respiratory (0%) and soft tissue and wound samples (2%) [16,36]. Yet, in a study by Hendrix *et al.*, 13% of all hospital patients who provided an oropharyngeal (back of the oral cavity) culture had VRE-positive results [37]. Rectal swabs are often used to detect VRE, however that type of sampling is very intrusive and was ruled out in order to increase participation among our study population. Using only nasal and dermal swabs to detect VRE and VSE from participants in the current study could have underestimated the true prevalence of VRE and VSE among the study population. In addition, participants in the study were not asked about antibiotic use in the months before the study and limited health-related data was collected. This type of information could have further contributed to understanding the potential human health risks from exposure to reclaimed water.

#### 3.2.3. Public Health Implications

Although we found no statistically significant differences in the odds of *S. aureus* or enterococci colonization between reclaimed water spray irrigation workers and office worker controls, the higher percentage of spray irrigation workers colonized with MDR MSSA and VSE raises potential public health concerns for those working with or otherwise exposed to reclaimed water. Several previous studies have analyzed the risk of different types of infections among individuals occupationally, recreationally, or residentially exposed to reclaimed water that has undergone various levels of treatment and found conflicting results [4,5]. A study in Mexican agricultural communities found increased odds of parasitic infections and associated diarrheal disease among individuals exposed to wastewater stored in one reservoir (which could be categorized as secondary (biologically) treated wastewater) and used for agricultural irrigation [4]. With additional time or storage in more than one reservoir, there was no difference in the odds of infection between exposed and unexposed groups [4]. Durand and Schwebach examined whether individuals recreationally exposed to turf irrigated with reclaimed water in Colorado were more likely to report gastrointestinal (GI) illnesses than those exposed to turf irrigated with potable water [6]. They found no difference in the number of reported illnesses between the exposed and unexposed groups. However, their study did show that exposure to wet grass was associated with more reported GI symptoms [6]. Because reclaimed water irrigation workers routinely work in wet grass as they are spraying, they could be at a greater risk for experiencing GI symptoms. Similarly, a study by Devaux *et al.* in France identified that farmers exposed to wastewater treated in stabilization ponds reported more respiratory and GI symptoms compared to a non-exposed group of controls [5]. A Texas-based study found that individuals exposed to secondary treated reclaimed water used for agricultural spray irrigation had an increased risk of viral infections 1.5–1.8 times that of individuals not exposed to reclaimed water [38].

To our knowledge, the current study is the first to evaluate occupational exposures to *S. aureus* and enterococci among workers using reclaimed water in the United States. Although the differences between the odds of MSSA, MDR MSSA, and VSE colonization among spray irrigators *vs.* controls in this study were not statistically significant, the data still provide evidence of potential human health issues that should be further investigated with larger sample sizes across the USA. This is particularly important with regard to potential exposures to MRSA—a leading cause of hospital-acquired infections and a microorganism associated with a growing number of community-acquired infections—because MRSA has been detected in treated U.S. wastewater that is used in reclamation activities [7]. Our research group also has identified VRE, vancomycin-intermediate resistantenterococci, and MSSA in reclaimed water [7,12]. Similarly, more work is needed to evaluate potential exposures to other human pathogens among reclaimed water spray irrigation workers including *Legionella* spp. and *Aeromonas* spp. since these waterborne microorganisms have also been isolated from reclaimed water [3,39,40].

## 4. Conclusions

Our findings suggest that the odds of MSSA, multidrug-resistant MSSA, and either MSSA or VSE colonization between spray irrigation workers using reclaimed water and those who are not routinely exposed to reclaimed water are not statistically significantly different. However, the lack of statistically significant findings could be an artifact of the limited number of spray irrigation workers available to participate in the study. As reclaimed water use continues to grow, additional studies with larger samples sizes are needed to further evaluate occupational exposures to human pathogens originating from this water source. 
